# A protocol and training guidelines for mosquito sampling in remote areas with limited power supply

**DOI:** 10.1016/j.mex.2024.102563

**Published:** 2024-01-09

**Authors:** Song-Quan Ong, Mahadimenakbar Mohamed Dawood, Homathevi Rahman, Mohd Farid Alias, Mohd Arshil Moideen, Ping-Chin Lee, Jodi M Fiorenzano, Nathaniel Christy, Thomas McGlynn, Noel Cote, Andrew G. Letizia

**Affiliations:** a Institute for Tropical Biology and Conservation, Universiti Malaysia Sabah, Malaysia; b Malaysian Armed Forces (MAF), Malaysia; c Biotechnology Research Institute, Universiti Malaysia Sabah, Jalan UMS, Kota Kinabalu, Sabah 88400, Malaysia; d Faculty of Science and Natural Resources, Universiti Malaysia Sabah, Jalan UMS, Kota Kinabalu, Sabah 88400, Malaysia; e U.S. Naval Medical Research Unit INDO PACIFIC, Singapore; f Vysnova Partners, LLC, Alexandria, VA 22314, USA

**Keywords:** Zoonotic, Malaria, Dengue, Anopheles leucosphyrus, Aedes, Plasmodium knowlesi, Chikungunya, Borneo, Malaysia, Zika, A protocol for sampling mosquitoes for sylvatic studies in hard-to-reach and power supply limited areas

## Abstract

Mosquito-borne diseases pose a significant threat in many Southeast Asian countries, particularly through the sylvatic cycle, which has a wildlife reservoir in forests and rural areas. Studying the composition and diversity of vectors and pathogen transmission is especially challenging in forests and rural areas due to their remoteness, limited accessibility, lack of power, and underdeveloped infrastructure. This study is based on the WHO mosquito sampling protocol, modifies technical details to support mosquito collection in difficult-to-access and resource-limited areas. Specifically, we describe the procedure for using rechargeable lithium batteries and solar panels to power the mosquito traps, demonstrate a workflow for processing and storing the mosquitoes in a -20 °C freezer, data management tools including microclimate data, and quality assurance processes to ensure the validity and reliability of the results. A pre- and post-test was utilized to measure participant knowledge levels. Additional research is needed to validate this protocol for monitoring vector-borne diseases in hard-to-reach areas within other countries and settings.

## Graphical abstract

**Figure:**
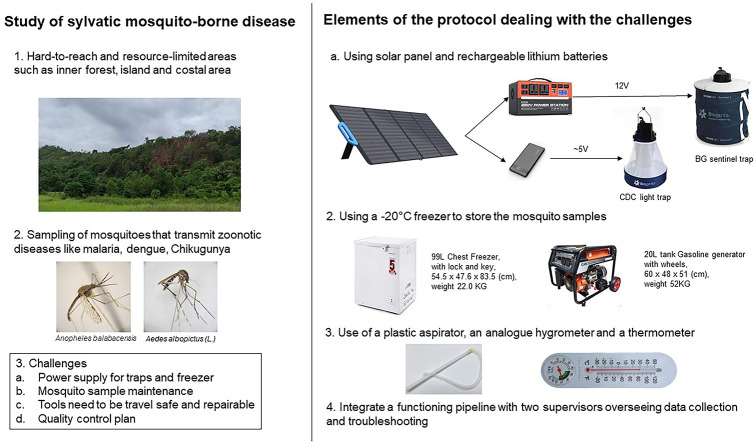

Specifications table

**Table:** 

Subject area:	Medicine and Dentistry
More specific subject area:	Mosquito-borne disease surveillance program
Name of your protocol:	A protocol for sampling mosquitoes for sylvatic studies in hard-to-reach and power supply limited areas
Reagents/tools:	1. Mosquito traps - CDC trap and BG -Sentinel trap
	2. Lithium batteries (5 V output)
	3. Lithium batteries (12 output)
	4. Chest freezer
	5. Plastic aspirato
	6. Analogue hygrometer/thermomete
	7. Solar panel (100 W)
	8. Gasoline generator (1000 W)
Experimental design:	This study describes a protocol for public health practitioners and medical entomologists to trap and collect mosquito samples in areas with extremely limited resources. Therefore, a protocol was created for collecting mosquitoes that includes a list of robost equipment that can be adapted to the environment. We designed the protocol to work in austere field environments where there is no electricity, difficult access, and limited resources. For the power supply, we used rechargeable lithium batteries and solar cells to power the traps. As the area is difficult to access, customized tools were proposed that are safe and repairable, such as a plastic aspirator, and a -20 °C freezer to store samples for pathogen screening. We also describe a workflow to train staff for timely implementation and quality control
Trial registration:	Not applicable
Ethics:	This project was approved by the Malaysian Ministry of Health (NMRR ID -23-00934- TOM) and the Universiti Malaysia Sabah Ethics Committee [JKEtika 3/23(13)]. All volunteers who performed the mosquito collections gave informed consent and received malaria prophylaxis throughout the study period.
Value of the Protocol:	• Describes mosquito collection procedures in areas that are difficult to access and lack electricity.
	• Demonstrates how rechargeable lithium batteries can be used in the field setting to operate mosquito traps and the methods for recharging the batteries with a solar panel in a jungle setting.
	• Truncates necessary training modules from two weeks to one saving costs and expediting sampling

## Description of protocol

### Rationale & background information

Sylvatic cycles of mosquito-borne diseases pose a significant threat to many Southeast Asian countries [1]. This is primarily due to the disturbance of human activities in forest areas such as logging and hunting, which increase exposure and risk to the human host [2]. Therefore, effective mosquito surveillance programs are essential to understand the distribution, ecology, and diversity of sylvatic vectors that bridge transmission where humans and forest areas overlap [3,4]. However, due to the remoteness of these locations, mosquito sampling is costly, inconvenient, and difficult to perform. The World Health Organization (WHO) and government agencies have established a framework and guidelines for mosquito sampling, but implementation in hard-to-reach forest areas with limited resources remains challenging. Therefore, mosquito-borne disease surveillance in such an area requires a protocol that meets some key criteria that are not addressed by the WHO protocol. These include: (1) power supply for mosquito traps; (2) ability to store mosquitoes for pathogen screening; (3) expeditious training time for sampling personnel; (4) safe, portable, and feasible tools; (5) quality assurance management. Our aim was to develop a protocol that addressed these criteria and included both cognitive (knowledge and theory-based) and psychomotor (hands-on) elements, mosquito collection techniques procedures, addressed power supply concerns, mosquito sample handling, basic identification, and data management. This manuscript is divided into two parts describing the protocol itself and the implementation and validation in Malaysia. The “Protocol Design” section describes the three main parts that are required for successful implementation – cognitive learning, psychomotor learning, and quality assurance. The “Validation of the protocol” section reviews and describes the results of the protocol after use in a training workshop. To increase the reusability of the protocol, the specifications of all materials have been included as Appendix 1 and the pre- and post-survey questions as Appendix 2.

### Protocol design

**Fig. 1:**
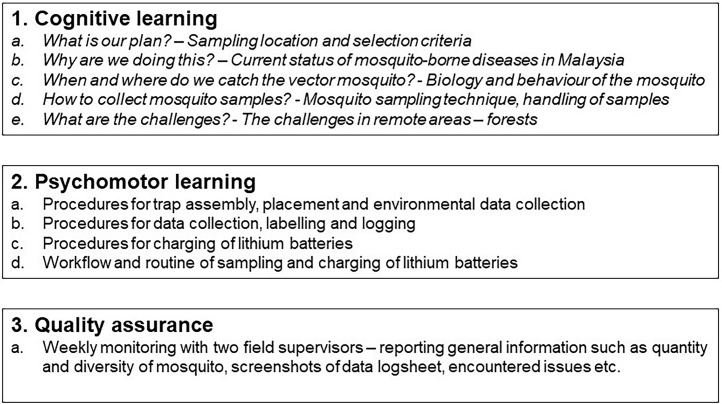
Three main parts of the protocol.

The training was designed based on the standard training guidelines of the World Health Organisation (WHO) [5,6]. We refer to the learning units – “Introduction to Medical Entomology”, “Identification of Dengue and Malaria Vectors” and “Sampling of Mosquitoes” and include the procedures for collecting mosquitoes in hard-to-reach areas with limited power supply Fig. 1. illustrates the three main parts of this protocol, which consists of cognitive learning covering knowledge about mosquito-borne diseases and mosquitoes, psychomotor learning focusing on the practical skills of assembling and placing traps, charging lithium batteries and data management, and quality assurance to ensure the validity and reliability of the results.

The first part of cognitive learning comprises the theoretical knowledge required to answer the study question below:

1. What is the location and plan for mosquito sampling?
2. Why do we collect mosquitoes?
3. When and where do we catch mosquitoes?
4. How do we collect mosquitoes?
5. What are the challenges and how do we overcome them?

**Fig. 2:**
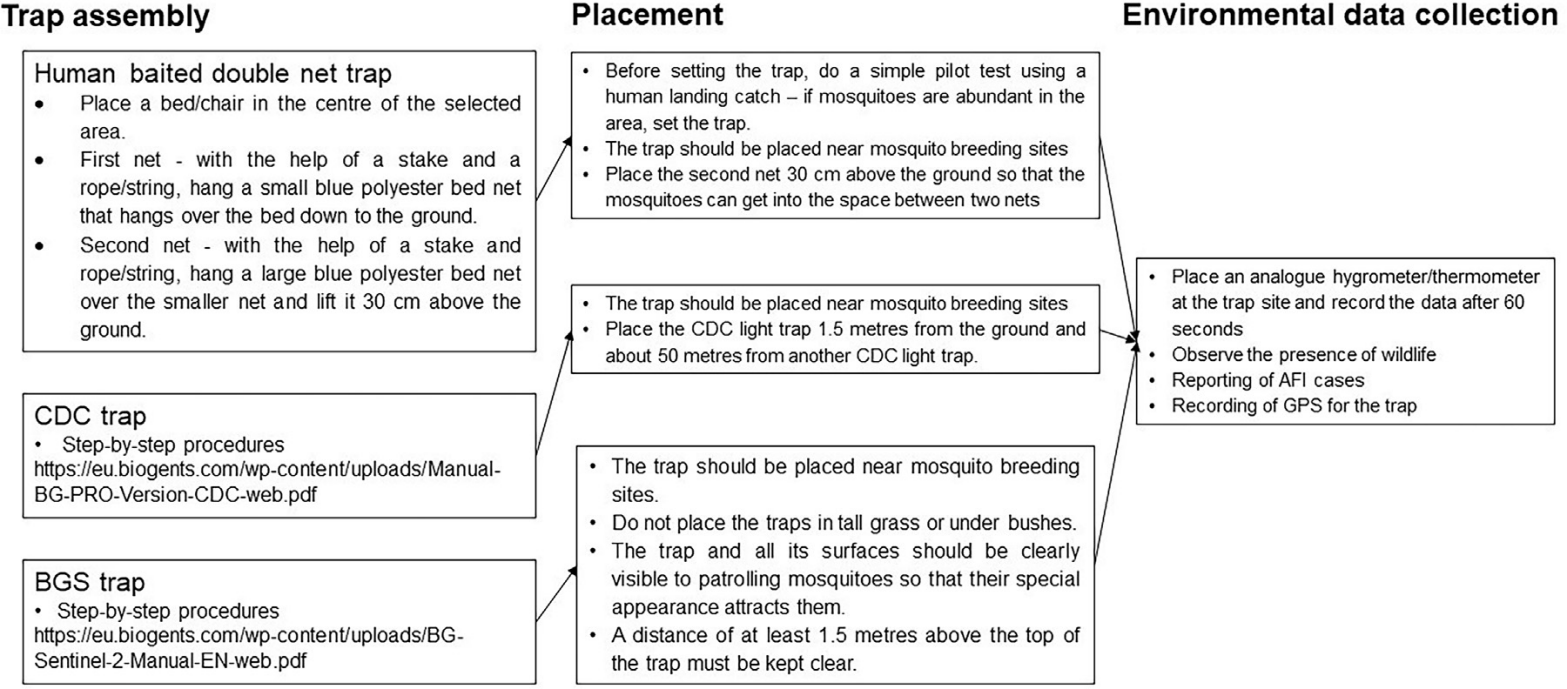
Procedure for trap assemble placement and environmental data collection.

**Fig. 3:**
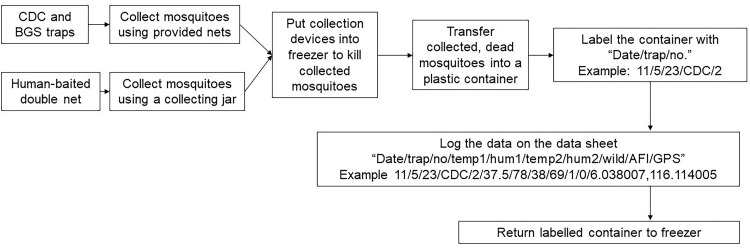
Procedures for data collection, label, and logging.

**Fig. 4:**
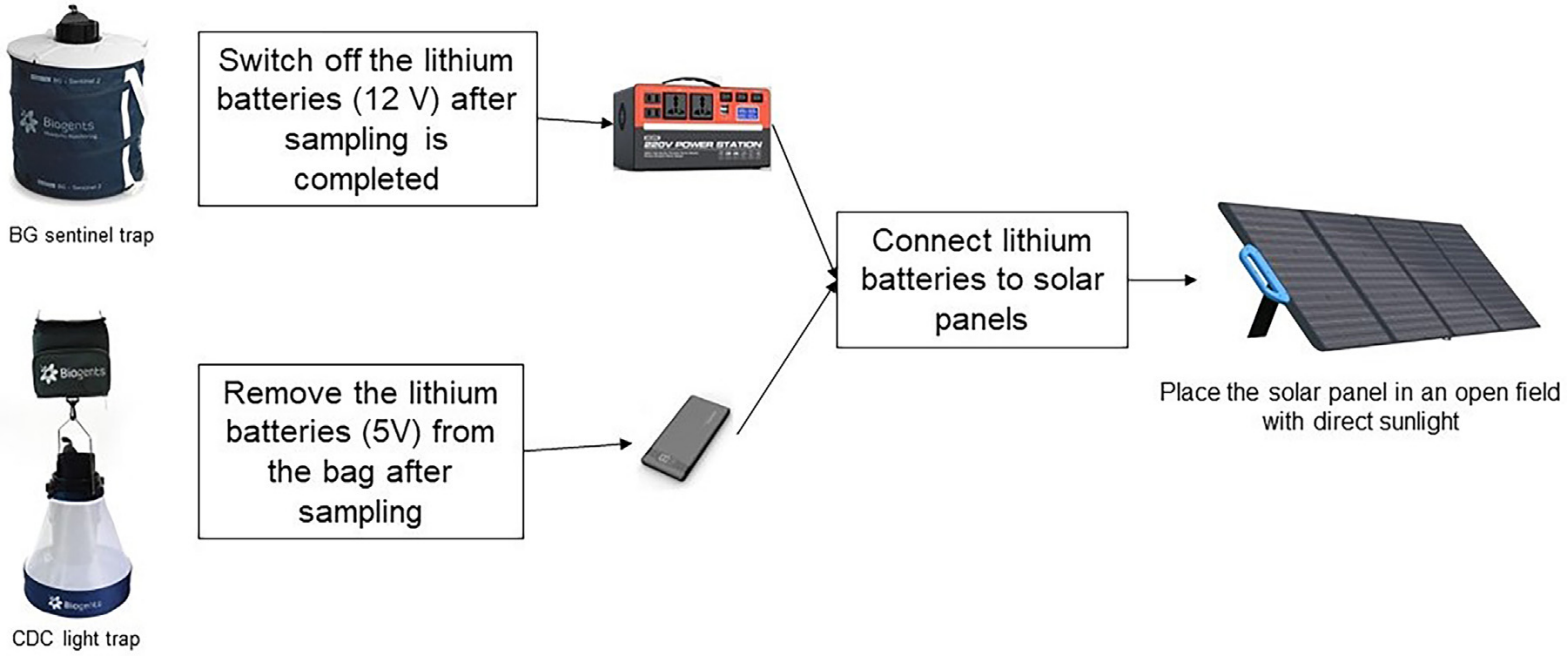
Procedures for charging of lithium batteries.

This knowledge transfer module and classroom learning and was primarily done through lectures and presentation slides as well as interactions with the participants through group discussions. The specific areas for group discussion included mosquito-borne diseases, the mosquito as a vector, and the theory behind specific traps. Later, the protocol describes psychomotor learning that focused on the practical procedures and workflows involved in preparing the trap, collecting the mosquitoes, processing, and storing the mosquitoes and managing the data. The psychomotor learning can be further divided into four subsections - procedures for trap assembly, placement, and environmental data collection (Fig. 2); procedures for data collection, labelling and logging (Fig. 3); procedures for lithium battery charging (Fig. 4); and the workflow and routine of sampling and lithium battery charging*.*

This protocol adopted specific units from the WHO [5,6] and the Pan American Health Organisation (PAHO) [7]. We emphasised hands-on training on mosquito collection and sample care and used technologies such as solar batteries and panels to support mosquito sampling in remote areas with limited electricity supply. The content selection was based on the vector index (VI) proposed by Centers for Disease Control and Prevention (CDC) [8], which emphasised vector sample collection, and the Malaysian Minister of Health [9], which stated that adult mosquitoes are the most important indicators for vector pathological surveillance investigation. Therefore, this protocol focused on adult mosquitoes. This protocol was tailored for personnel, such as military personnel and public health officials, deployed in a remote area that has sufficient sunlight (due to the use of solar panels to power the trap and freezer) to collect and care for mosquitoes adequately until further analysis in the laboratory can be executed. The training manual developed by PAHO [7] included a total of 10 units, ranging from community education to larval collection and care, control and prevention, and insecticide resistance. The training module on malaria published by WHO [5] has a total of 7 units, including malaria vector control, insecticide resistance management and the epidemiological strata of malaria. Both of these modules or protocols require at least 4 weeks of training. Our proposed protocol can be completed in one week as it emphasises hands-on training of mosquito sampling. This was achieved by excluding the units on population education, larval sampling and colony maintenance, vector control and prevention approaches, insecticide resistance and epidemiological modelling. The reason for the exclusion of these units was mainly that the protocol focused on the details of mosquito collection and storage of samples at low temperature (cold chain) and that control measures, insecticide resistance, epidemiological modelling, etc. were not relevant and useful for the personnel used for sampling in the field. The exclusion of all these issues needs to be supported by a team in a laboratory focused on pathogen screening and modelling.

**Fig. 5:**
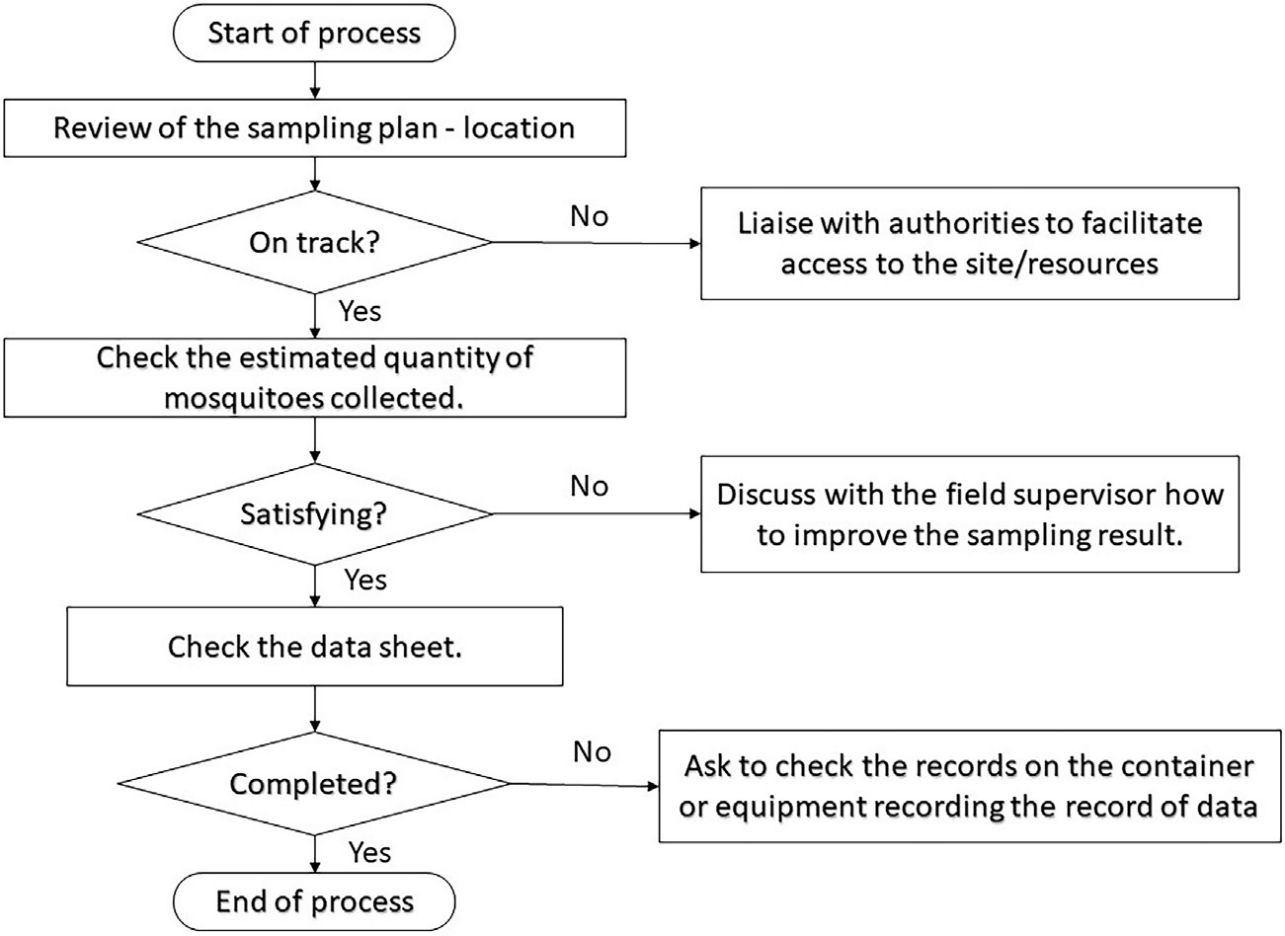
Flowchart of quality assurance of the protocol.

The third part of the protocol is quality assurance, which consists of a flow chart as described in Fig. 5. This process is securely managed by two supervisors, one of whom works on-site with the sampling staff and oversees the process of data collection, logging, and sample maintenance, while the other is remotely responsible for technical support, easy identification of mosquitoes, replenishment of consumables and logistical planning from one site to another.

### Validation of the protocol

**Fig. 6:**
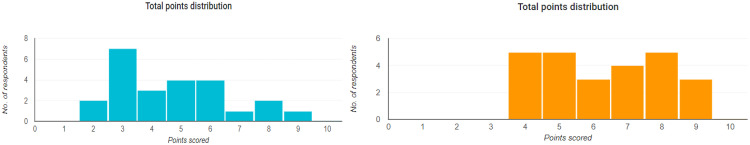
The results of the pre- and post-course surveys. Left, pre-course surveys (*n* = 25, mean = 4.71 ± 0.08); Right, post-course surveys (*n* = 25, mean = 6.32 ± 0.07).

To validate the protocol, a training workshop was held at the Malaysian Army Combat Centre in Ulu Tiram Johor Malaysia where zoonotic malaria cases were reported. The sampling site was located about 10 km from the urban area of Ulu Tiram, a suburb that is mostly covered by forest or vegetation. A total of 25 participants from different states in Malaysia attended the workshop, including medical laboratory technicians, eight medical officers, and eight public health practitioners. As described in the protocol, the workshop was designed according to the three parts of the protocol as previously described. At the start of the training, the objectives of the workshop were explained, followed by a lecture on the content of the guide, followed by a practical exercise in which the acquired knowledge was applied with the help of the trainer. In the practical portion of the training, the participants had to assemble and set up traps in the field, collect mosquitoes and process the samples with the logged data. A survey was conducted before and after the workshop to assess the participants' skills and knowledge. In particular, participants had to answer questions on the following topics: (1) What is a mosquito-borne disease? (2) How do you collect mosquito samples in a remote area and how do you maintain the samples’ condition? (3) How do you determine the taxonomy of mosquitoes? Appendix 2 shows the ten questions that used for pre and post survey. For psychomotor learning, participants needed to demonstrate how to properly assemble the BG Sentinel, BG Pro, CDC Light and Human-baited double net traps, place these trap correctly, handle the mosquito samples, charge the batteries with a solar panel and use an illustrated key to determine mosquito taxonomy, and the assessment was carried out by two medical entomologists (second and third author of this article) who have at least 10 years of experience in this field. . The pre- and post-surveys were evaluated using 10 questions (Appendix 1) and validated by a pilot test with a group of 6 students (with a bachelor's degree and in postgraduate studies). The results of the pre- and post-course surveys were normally distributed (Shapiro-Wilk Normality Test *W* = 0.096, *p* = 0.25), and the results of the post-course surveys (*n* = 25, mean ± standard error, 6.32 ± 0.07) were significantly higher than the results of the pre-course surveys (*n* = 25, mean ± standard error, 4.71 ± 0.08) at *p* < 0.05 by a paired *t*-test (Fig. 6). This indicates that the workshop was able to improve participants' knowledge and skills on vector mosquitoes, mosquito-borne diseases, sampling methods and identification.

## Discussion

The most important contributions of the protocol were the emphasis on hands-on training, the use of solar technology, the customised approach for remote areas and the shortened training period. Together, these aspects contribute to an improved and more accessible mosquito trapping protocol for resource-limited areas and ultimately improve vector pathological surveillance in areas with limited resources such as electricity. Like many previous attempts at insect trapping with solar-powered devices [10,11], the solar panel allow recharging of the lithium batteries that used to power the fan and lights of traps. However, the practical use of portable solar-powered devices for mosquito trapping is still very new and, to our knowledge, has not been widely reported. For example, the solar panels and batteries used in this study were fully portable, allowing staff to move sampling from one location to another, unlike other studies that used passive traps with a static solar station [11]. In addition, this protocol has been tailored to remote areas with limited electricity, where the mosquito samples cannot be stored in an ethanol solution like other insects or animals [12,13] but require a freezer at -20 °C to preserve the nucleic acid content of the pathogen in the vector for further analysis. To teach all these technical procedures in conjunction with the limited time available to staff, a shortened training duration of one week was proposed and demonstrated, excluding non-essential modules. The rationale for excluding modules could allow for more focussed and practical training specifically designed for resource-constrained field environments.

Nevertheless, the protocol also had some potential limitations. The first was the reliance on the reliability of solar power, which is a potential challenge in areas with unpredictable weather conditions or limited sunlight. Nonetheless, in our protocol, a petrol-powered electricity generator served as a backup power supply in case the solar batteries could not be fully charged due to weather conditions. The second limitation was the exclusion of essential modules such as larval collection, vector control and prevention approaches, and insecticide resistance, which could be crucial for comprehensive mosquito surveillance and control. However, we also offer additional workshops for staff who want to learn more than just the protocol to address broader vector-borne disease control efforts and comprehensive public health measures. In conclusion, the outcome of the survey and mosquito collection shows that the manual can be used given the limited time and resources, especially the power supply and logistical plan. The manual could serve as a basis for future capacity building training on sampling, sample handling and mosquito identification in rural and forested areas to optimise sampling and pathogen screening results.

## Conclusion

In conclusion, the proposed protocol for mosquito sampling was a valuable tool for researchers and public health practitioners working in off-grid and remote areas. By integrating targeted learning modules, robust sampling methods and solar-powered devices, we support surveillance in areas where traditional approaches may not be sufficient and provide a pathway to more comprehensive and accurate disease risk assessments.

## Author disclaimer

The views expressed in this article reflect the results of research conducted by the author and do not necessarily reflect the official policy or position of the Department of the Navy, Department of Defense, nor the United States Government.

All authors declare no competing or conflicts of interests. The views expressed in this article reflect the results of research conducted by the author and do not necessarily reflect the official policy or position of the Department of the Navy, Department of Defense, nor the United States Government. NC (LCDR, MSC, USN), AL (CAPT, MC, USN) are military service members. This work was prepared as part of my official duties. Title 17U.S.C. 105 provides that `copyright protection under this title is not available for any work of the United States Government.' Title 17U.S.C. 101 defines a U.S. Government work as work prepared by a military service member or employee of the U.S. Government as part of that person's official duties.

## CRediT authorship contribution statement

**Song-Quan Ong:** Conceptualization, Methodology, Validation, Formal analysis, Investigation, Resources, Data curation, Writing – original draft, Writing – review & editing, Visualization, Project administration. **Mahadimenakbar Mohamed Dawood:** Conceptualization, Methodology, Validation. **Homathevi Rahman:** Conceptualization, Methodology, Validation. **Mohd Farid Alias:** Conceptualization, Methodology, Validation, Supervision. **Mohd Arshil Moideen:** Conceptualization, Methodology, Validation, Supervision. **Ping-Chin Lee:** Formal analysis, Investigation, Resources. **Jodi M Fiorenzano:** Conceptualization, Validation, Methodology. **Nathaniel Christy:** Validation, Supervision, Project administration. **Thomas McGlynn:** Conceptualization, Methodology, Validation. **Noel Cote:** Methodology, Validation. **Andrew G. Letizia:** Validation, Supervision, Project administration.

## Declaration of Competing Interest

The authors declare that they have no known competing financial interests or personal relationships that could have appeared to influence the work reported in this paper.

## Data Availability

Data will be made available on request.

## Appendix Supplementary materials

Image, application 1

Image, application 2

## References

[1] Valentine M.J., Murdock C.C., Kelly P.J. (2019). Sylvatic cycles of arboviruses in non-human primates. Parasites Vectors.

[2] Weaver S.C., Barrett A.D.T. (2004). Transmission cycles, host range, evolution and emergence of arboviral disease. Nat. Rev. Microbiol..

[3] Hanley K.A., Monath T.P., Weaver S.C., Rossi S.L., Richman R.L., Vasilakis N. (2013). Fever versus fever: the role of host and vector susceptibility and interspecific competition in shaping the current and future distributions of the sylvatic cycles of dengue virus and yellow fever virus. Infect. Genet. Evol..

[4] Vasilakis N., Cardosa J., Hanley K.A., Holmes E.C., Weaver S.C. (2011). Fever from the forest: prospects for the continued emergence of sylvatic dengue virus and its impact on public health. Nat. Rev. Microbiol..

[5] World Health Organization. (2013). Malaria entomology and vector control

[6] World Health Organization. (2009). Dengue: guidelines for diagnosis, treatment, prevention and control. World Health Organization 23762963

[7] Williams J., Pinto J. (2012).

[8] Centers for Disease Control and Prevention (CDC) (2023) mosquito: environmental Surveillance. Accessed on 1 November 2023. https://www.cdc.gov/mosquitoes/guidelines/west-nile/surveillance/environmental-surveillance.html#:~:text=The%20Vector%20Index%20(VI)%20estimates,individual%20species%20(Gujral%20et%20al.

[9] Ali R., Ali W.N.W.M., Putit P.W. (2023). Updating the data on malaria vectors in Malaysia: protocol for a scoping review. JMIR Res. Protoc..

[10] Balamurugan R., Kandasamy P. (2021). Effectiveness of portable solar-powered light-emitting diode insect trap: experimental investigation in a groundnut field. J. Asia Pac. Entomol..

[11] Ramesh D., Chandrasekaran M., Soundararajan R.P., Subramanian P.P., Palled V., Kumar D.P. (2022). Solar-powered plant protection equipment: perspective and prospects. Energies.

[12] Marquina D., Buczek M., Ronquist F., Łukasik P. (2021). The effect of ethanol concentration on the morphological and molecular preservation of insects for biodiversity studies. PeerJ.

[13] Astrid T., Margit E., Leopold F. (2016). Ethanol: a simple and effective RNA-preservation for freshwater insects living in remote habitats. Limnol. Oceanogr. Methods.

