# Supplementary material for: A protocol and training guidelines for mosquito sampling in remote areas with limited power supply
Source: MethodsX. 2024 Jan 9;12:102563. doi: 10.1016/j.mex.2024.102563 (PMC10847759; doi:10.1016/j.mex.2024.102563)
Supplement: Supplementary file 1 [file mmc1.docx]

Appendix I

| **Items** | **Brand and model** |
| --- | --- |
| CDC trap | Biogents, Biogents Pro |
| BGS trap | Biogents, Biogents Sentinel |
| Lithium rechargeable batteries (for CDC trap) | PENENG, PN-852PD 10000mAh PD/QC 3.0 20W Built-In Cables Power Bank |
| Lithium rechargeable batteries (for BGS trap) | VANPA, Vanpa Portable Power Station 400W AC220V Output, capacity 180000mAh |
| Solar panels | VANPA, VANPA 100W Foldable Solar Panel |
| Generator | Ogawa, GW3100Watt/3.1kW Professional Manual/Electric Start Gasoline Generator |
| Freezer (100L, -20C) | Haier, Chest Freezer 100L BD138HMC |
