# Supplementary material for: A protocol and training guidelines for mosquito sampling in remote areas with limited power supply
Source: MethodsX. 2024 Jan 9;12:102563. doi: 10.1016/j.mex.2024.102563 (PMC10847759; doi:10.1016/j.mex.2024.102563)
Supplement: Supplementary file 2 [file mmc2.pdf]

Appendix II

Pre-course assessment - mosquito sampling/ID

This is an post- course evaluation for the mosquito sampling and ID training workshop that organised in PULADA Johor from 6 to 10 February 2023

* Indicates required question

1. Email *

____

2. What is **NOT** the role of an medical entomologist in the control of mosquito-borne diseases? * 1 point

Mark only one oval.

- ⬭ Mosquito population monitoring/mosquito surveillance
- ⬭ Predicting dengue transmission based on risk factors - help in decision-making
- ⬭ Clean the breeding places of mosquitoes in the household
- ⬭ Evaluate the effectiveness of a mosquito control approach/program

3. The mosquito goes through a complete metamorphosis, i.e. how many stages of the life cycle does it consist of? * 1 point

Mark only one oval.

- ⬭ 1
- ⬭ 2
- ⬭ 3
- ⬭ 4
- ⬭ 5

4. One of the successes of the mosquito is that immature and adult do not share the same habitat. What is the habitat of the immature mosquito? 1 point

Mark only one oval.

- Vegetation
- Aquatic
- Natural container
- Artificial container

5. Sampling of mosquitoes can be carried out using the following methods, except for 1 point

Mark only one oval.

- Human Landing Catch (HLC)
- Human baited double net (HBDN)
- Physical trap - BGS, CDC ovitrap
- Hand picking

6. Larval indices are recommended by WHO for vector surveillance, but are controversial when it comes to detecting disease transmission. What is the main reason for this? 1 point

Mark only one oval.

- Inconsistence outcome
- larval population is not representing the vector capacity
- larval population is not correlate with the adult vector mosquio
- larval population is too small to be used as the indices

7. Which of the following methods is so far the **MOST** effective for collecting Anopheles mosquitoes? 1 point

Mark only one oval.

- CDC
- BGS
- Ovitrap
- Human as bait

8. Adult mosquitoes collected by HLC or HBDN must be maintained for the following reason: 1 point

Mark only one oval.

- To extend the lifespan of pathogen in the mosquito
- To rear the colony
- To increase the population
- To conserve the mosquito

9. What is the BEST procedure to maintain adult mosquitoes? 1 point

Mark only one oval.

- Keep in separate container and at -20C freezer
- Keep in a normal fridge
- Keep in separate container at room temperature
- Keep in a plastic bag

10. Mosquitoes can be identified to species level using the morphology key. 1 point

The illustration below shows four insects. Which of them is a mosquito?

Mark only one oval.

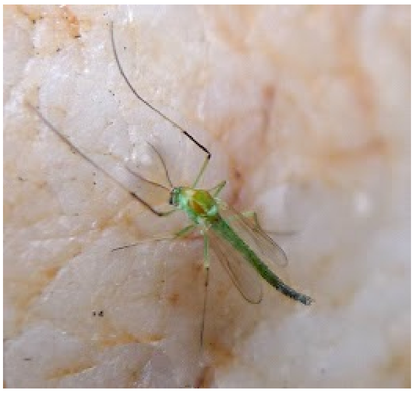

Option 1

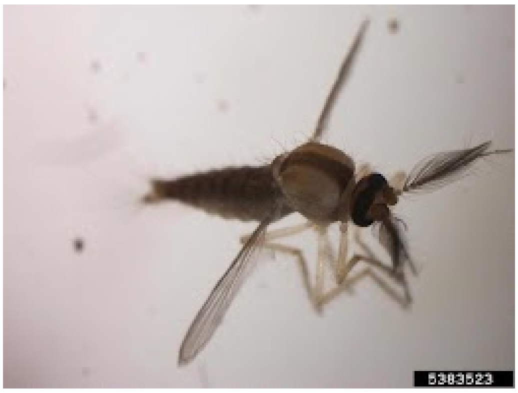

Option 2

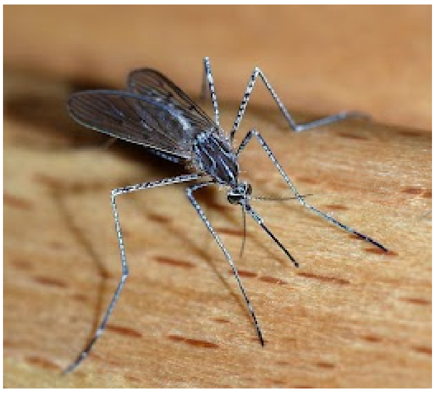

Option 3

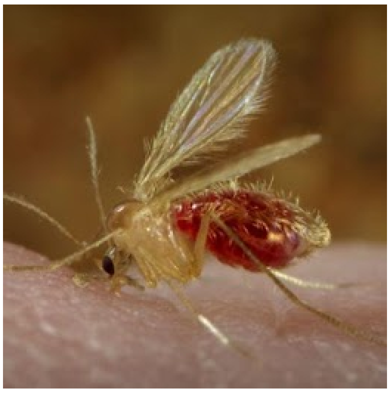

Option 4

Rueda's Pictorial Keys below shows the morphological distinction between *Aedes aegypti* and *Aedes albopictus*. Which of them is the *Aedes albopictus*?

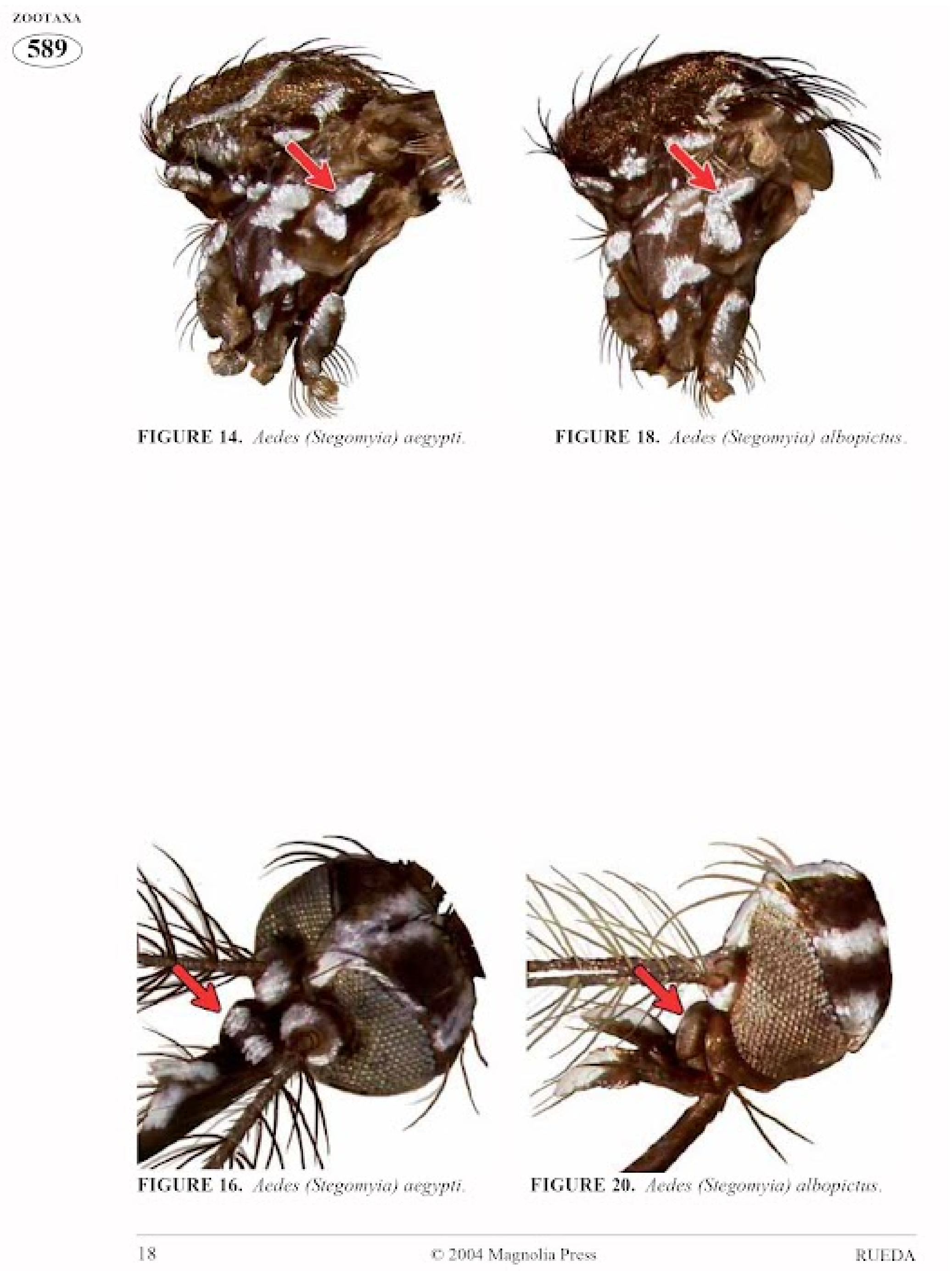
ZOOTAXA
589

FIGURE 14. *Aedes (Stegomyia) aegypti.* FIGURE 18. *Aedes (Stegomyia) albopictus.*

FIGURE 16. *Aedes (Stegomyia) aegypti.* FIGURE 20. *Aedes (Stegomyia) albopictus.*

18 © 2004 Magnolia Press RUEDA

11. Aedes aegypti or Aedes albopictus? * 1 point

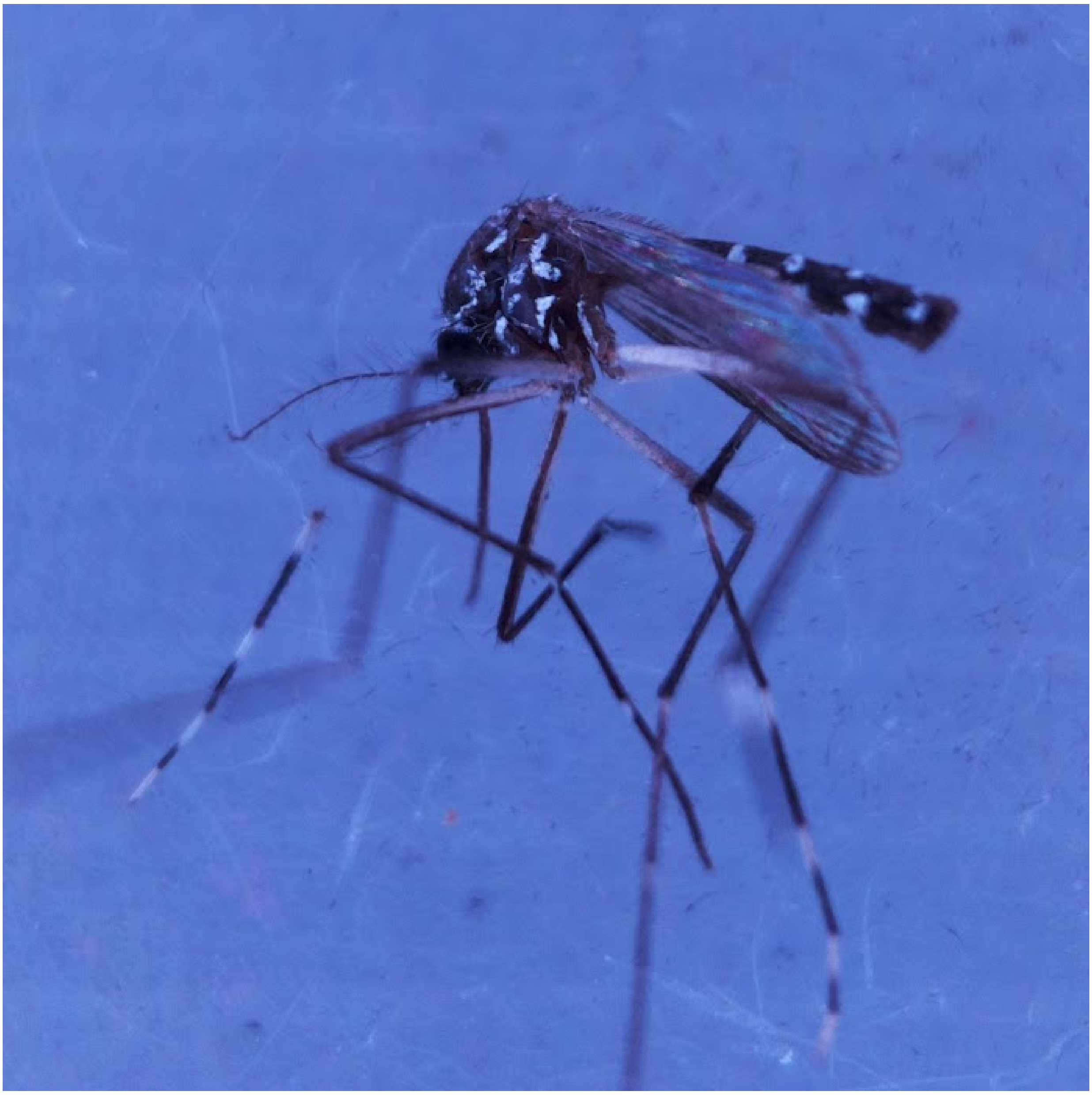

Mark only one oval.

- Aedes aegypti
- Aedes albopictus

This content is neither created nor endorsed by Google.

Google Forms
